# Multimodal risk profiles reveal shared and disease-specific risks of major non-communicable diseases: a prospective cohort study of 42,666 individuals 

**DOI:** 10.3389/ijph.2026.1609591

**Published:** 2026-07-09

**Authors:** Qiaoyi Xu, Zhenqiu Liu, Renjia Zhao, Zixuan Cui, Xufei Xing, Xingdong Chen, Chen Suo

**Affiliations:** 1 School of Public Health, Fudan University, Shanghai, China; 2 Taizhou Institute of Health Sciences, Fudan University, Taizhou, China; 3 Human Phenome Institute, Fudan University, Shanghai, China; 4 Shanghai Institute of Infectious Disease and Biosecurity, Fudan University, Shanghai, China

**Keywords:** blood biomarkers, cohort study, multi-disease risk stratification, non-communicable diseases, risk stratification

## Abstract

**Objectives:**

Evaluate whether circulating blood biomarker profiles identify shared and disease-specific risks of non-communicable diseases (NCDs).

**Methods:**

We considered 42,666 participants from Taizhou, China. After exclusions, discovery (n = 14,478; recruited 2011.9–2014.1) and temporal validation (n = 25,018; recruited 2018.7–2021.11) cohorts were defined. We integrated 54 blood biomarkers and 26 questionnaire/physical indicators. Predictors were selected after Cox pre-screening based on concordant inclusion across stepwise regression, regularized regression, and Boruta random forest.

**Results:**

The final score included 15 biomarkers plus age, smoking, hypertension, and vegetable intake. In the temporal validation cohort, high-risk individuals (25.0% of participants) accounted for 53.9% of incident major NCD cases, with a 6.29-fold (4.83–8.19) higher risk than the low-risk group. Similar gradients were observed for all-cause mortality. Compared with disease-specific scores, the combined score effectively stratified both composite and individual outcomes and revealed shared risks: 56.1% of disease-specific high-risk individuals were also high risk for other NCDs.

**Conclusion:**

A score integrating blood biomarkers with epidemiological and physical measures achieved clear risk stratification in the temporal validation cohort and may support priority population identification for major NCDs.

## Introduction

Non-communicable diseases (NCDs) represent a primary global public health threat, accounting for 74% of total global deaths. In 2023, China recorded approximately 9.55 million NCD-related deaths, accounting for 91% of all deaths. Among NCD-related deaths, cardio-cerebrovascular diseases (CVD), cancers, and chronic respiratory diseases (CRD) are leading contributors, collectively accounting for 80% of these deaths [1]. Many major NCDs are preceded by long-term risk accumulation, making early identification and risk stratification important for prevention and population health management [2]. This underscores the need for accessible risk assessment approaches that can be embedded into routine health examinations.

Risk stratification helps identify high-risk individuals before clinical onset and supports preventive action. However, assessing risks across multiple outcomes often requires collecting many predictors or conducting disease-specific assessments separately, which can be cumbersome and difficult to scale. Using data generated in comprehensive health examinations may therefore be a more practical route to multi-outcome assessment.

With the increasing popularity of health examinations, blood-based biomarker studies have increasingly shown potential for disease risk assessment and long-term health risk identification [3–5]. A single examination typically yields dozens of blood indicators, yet current reports mainly evaluate whether each biomarker falls within its reference range and rarely provide an integrated interpretation of future health risks. Blood indicators collectively reflect the body’s metabolic, inflammatory, hepatic, renal, and immune status, and their overall pattern, referred to as the blood profile, may serve as an early signal for future chronic disease risks and overall health outcomes such as mortality [6–9]. Yet in real-world health examination settings, evidence remains limited on whether such blood profiles can prospectively indicate multiple long-term outcomes.

Many existing studies focus on single outcomes, and evidence for longitudinal multi-outcome risk stratification using richer health-examination blood profiles is still evolving. Internationally, large prospective cohorts such as the UK Biobank have advanced risk assessment for multiple diseases and mortality using multi-modal frameworks, biomarker-based scores, and omics profiles [10–12]. However, models requiring high-dimensional omics, imaging, genetic, or detailed clinical data may not always be directly implementable in routine health examination settings. In addition, many existing biomarker-based models mainly include hematological and biochemical markers, whereas fewer incorporate broader markers related to tumor, infection, and organ function.

To address this challenge, this study uses data from the Taizhou Longitudinal Study (TLS), a large Chinese prospective cohort. By integrating 54 blood biomarkers with 26 questionnaire- and physical examination-derived indicators, we developed and temporally validated a composite risk score for major NCDs. By combining multiple indicators, this study seeks to move beyond the binary judgment of individual test results as “normal or abnormal” toward an integrated assessment of long-term health risks, providing methodological evidence for risk stratification and priority population identification in health examination settings.

## Methods

### Study design and participants

The TLS is a community-based cohort initiated in Taizhou, China since 2007 [13]. Among TLS participants considered for this analysis, 16,358 individuals enrolled from September 2011 to January 2014 and 26,308 individuals enrolled from July 2018 to November 2021 were initially considered for the discovery and temporal validation cohorts, respectively. After restricting to participants with available valid serum or plasma samples and applying further exclusions for baseline major NCD history, missing key data, or incident major NCDs within 180 days post-baseline, 14,478 and 25,018 participants were finally included, respectively. The study flowchart is presented in Supplementary Figure S1.

The study was approved by the Ethics Committee of Fudan University Taizhou Institute of Health Sciences (Ethics Approval No. B016) and was conducted in accordance with the World Medical Association Declaration of Helsinki. Written informed consent was obtained from all participants prior to enrollment in the TLS.

### Clinical biomarkers and covariates

Baseline data were collected via face-to-face interviews, physical examinations, and blood sampling. Peripheral blood samples (8–10 mL) were collected into K2 EDTA vacuum tubes, stored at 4 °C until same-day processing. After centrifugation to separate plasma, samples were aliquoted into barcoded cryovials and stored at −80 °C or below. Biochemical tests for both cohorts were centrally conducted at Shanghai Fourth People’s Hospital from June to September 2024 [14, 15], using consistent laboratory platforms and quality-control procedures. The study ultimately collected 54 circulating blood biomarkers and 26 questionnaire- and physical examination-derived indicators [16]. The latter covered demographic and socioeconomic factors, lifestyle factors, anthropometric and body composition factors, blood pressure-related factors, and dietary factors. Details of blood biomarkers are provided in Supplementary Table S1, candidate variable categories and their relevance to major NCD risk are summarized in Supplementary Table S2, and definitions, missing rates, and screening status of questionnaire- and physical examination-derived indicators are provided in Supplementary Table S3.

### Data preprocessing

Variables with >20% missingness in either cohort were excluded; missingness of blood biomarkers and questionnaire/physical indicators is summarized in Supplementary Tables S1, S3. For missing values, categorical variables were imputed with the most frequent category, continuous variables with the median. Except for *Helicobacter pylori* antibody, circulating blood biomarkers were standardized within each cohort and entered association models as continuous variables, with effect estimates expressed per 1-standard-deviation increase. For highly correlated biomarkers (Pearson’s correlation >0.8), the one with lower missing rate or higher variance was retained. After filtering by missingness, overlap, correlation, and Cox pre-screening, 25 blood biomarkers and 11 traditional factors were retained for multi-method selection.

### Outcome

The primary outcome was incident major NCDs (cancer, CVD, and CRD). All-cause mortality was treated as a secondary outcome to examine whether the derived risk score and risk groups also differentiated all-cause mortality risk. Disease outcomes were determined using International Classification of Diseases, 10th Revision (ICD-10) codes from the Taizhou Center for Disease Control and Prevention and local medical security systems; all-cause mortality was defined as death from any cause during follow-up and was ascertained through the same record linkages. Diagnoses were validated via pathological reports, imaging, or clinical assessments. Cancer outcomes included five common types: lung cancer (C33, C34), esophageal cancer (C15), gastric cancer (C16), liver cancer (C22), colorectal cancer (C18-C20). CVD included coronary heart disease (I20-I25) and stroke (I60-I64, I69); CRD was represented by chronic obstructive pulmonary disease (J41-J44).

For individuals with multiple major NCD diagnoses, only the earliest incident event is considered for the composite outcome; for disease-specific analyses, only the first recorded diagnosis within each disease category is retained. To reduce the influence of undiagnosed disease at baseline and ensure valid incident cases, only events occurring ≥6 months after baseline were included. Follow-up started 6 months after baseline and ended at the first target event, withdrawal, death, or the administrative censoring date of December 31, 2023, whichever came first.

### Development and validation of major NCDs identification model (NIMO)

In the discovery cohort, multivariable Cox proportional hazards regression was first used to screen candidate predictors. Least absolute shrinkage and selection operator (LASSO), stepwise regression, and Boruta random forest were then applied in parallel, and variables consistently selected across these three methods were retained for construction of the final NIMO score (Supplementary Figure S2). LASSO used cross-validation to select the penalty parameter; stepwise regression used bidirectional selection based on the Akaike information criterion; Boruta random forest was run with 500 trees and a maximum of 50 runs.

Variables were categorized into subgroups. Clinical reference limits or commonly used clinical thresholds were prioritized where appropriate; for variables lacking uniform clinical thresholds or with limited clinical discrimination, cut-offs were determined using the *maxstat.test* function in the discovery cohort. Cox model coefficients were then used to derive relative risk points for each subgroup, with the reference subgroup assigned 0 points. For interpretability, coefficients were multiplied by 10 and converted into point values. Total risk scores were summed across variables for each participant. Participants were classified into low-risk (<25th percentile), intermediate-risk (25th–75th percentile), and high-risk (>75th percentile) groups.

### Temporal validation and robustness analyses

All cut-offs were fixed when applied to the temporal validation cohort. The scoring rule derived from the discovery cohort was fixed and directly applied to participants recruited during a later period, allowing assessment of temporal reproducibility. Stratification performance was evaluated in both cohorts using cumulative incidence curves and hazard ratios for incident major NCDs; all-cause mortality was examined as a secondary outcome to assess whether the risk groups also differentiated mortality risk.

Robustness analyses included multiple imputation and complete-case analysis [17], alternative risk-group thresholds, alternative predictor-selection strategies, continuous z-standardized biomarker modeling, sex- and age-stratified analyses, non-CVD and subtype-specific outcomes to examine whether the composite-outcome findings primarily reflected CVD events, an expanded outcome definition including diabetes (E10–E14), breast cancer (C50), and asthma (J45–J46), glucose-excluded scoring, and a fixed 3-year follow-up window.

Internal robustness was assessed using 50 repeated 7:3 splits within the discovery cohort, stratified by incident major NCD status. In each split, the final predictors were retained, Cox coefficients were re-estimated in the training subset, and performance was evaluated in both subsets. Variable-selection stability was assessed by repeating the full predictor-selection procedure in 50 bootstrap resamples [18, 19].

### Comparison with disease-specific and information-source scores

Cancer-, CVD-, and CRD-specific scores were developed in the discovery cohort using the same candidate predictor pool, selection procedure, categorization strategy, and coefficient-based scoring framework as the NIMO score, with each first incident disease subtype as the target outcome. Within the discovery cohort, we compared high-risk overlap, stratification performance, and discrimination between the NIMO score and the disease-specific scores across composite and subtype outcomes.

To evaluate information-source contributions, two additional scores were developed using the same framework: a traditional-factor score based on questionnaire- and physical examination-derived variables, and a blood-based score based on circulating blood biomarkers plus age and sex. Both were developed in the discovery cohort, applied to the temporal validation cohort, and compared with the NIMO score using risk gradients, hazard ratios, and areas under the receiver operating characteristic curves (AUCs).

### Statistical analysis

Continuous variables are presented as mean ± standard deviation for approximately normally distributed data or median (interquartile range) otherwise. Continuous variables were compared using Welch’s t test or the Wilcoxon rank-sum test, as appropriate. Categorical variables were compared using the χ^2^ test or Fisher’s exact test. Risk stratification was evaluated using hazard ratios (HRs) and 95% confidence intervals (CIs). For biomarker-outcome association analyses, multivariable Cox proportional hazards models were adjusted for age, sex, body mass index, smoking status, alcohol drinking, hypertension, and daily vegetable intake. Discrimination was assessed using AUC, with the concordance index (C-index) reported where applicable. Differences in AUCs between scores were compared using the DeLong test.

A two-sided P value <0.05 was considered statistically significant. All analyses were performed using R software (version 4.3.2).

## Results

### Baseline characteristics and follow-up overview

The discovery cohort included 14,478 participants with 2,597 incident major NCD events and 632 deaths. The temporal validation cohort included 25,018 participants with 726 incident major NCD events and 213 deaths. The discovery cohort had a median follow-up duration of about 10.84 years, including 14,478 participants. In subtype-specific analyses, 543 cancer events, 1,601 CVD events, and 719 CRD events were identified. The temporal validation cohort had a median follow-up of 3.98 years; in subtype-specific analyses, 179 cancer events, 460 CVD events, and 120 CRD events were identified. Baseline characteristics of both cohorts are shown in Table 1 at the end of the text. Participants in the temporal validation cohort were older at baseline, whereas sex distribution was similar between cohorts.

**TABLE 1:** Baseline characteristics in the discovery and temporal validation cohorts (Taizhou Longitudinal Study, Taizhou, China, 2011–2023).

Characteristic	Discovery cohort		*P value*	Temporal validation cohort		*P value*
	N = 14,478			N = 25,018		
​	No incident major NCDs	Incident major NCDs	​	No incident major NCDs	Incident major NCDs	​
N, n (%)	11,881 (82.2)	2,597 (17.8)	​	24,292 (97.1)	726 (2.9)	​
Age, years	50.1 ± 8.9	56.3 ± 7.4	**<0.001**	53.1 ± 9.7	59.4 ± 6.7	**<0.001**
Sex, n (%)	​	​	**<0.001**	​	​	**<0.001**
Male	4,173 (77.7)	1,201 (22.3)	​	8,976 (96.2)	352 (3.8)	​
Female	7,708 (84.7)	1,396 (15.3)	​	15,316 (97.6)	374 (2.4)	​
Body mass index, kg/m^2^	24.5 ± 3.1	24.6 ± 3.3	**0.035**	24.8 ± 3.7	25.0 ± 3.5	0.112
Marital status, n (%)	​	​	**<0.001**	​	​	0.366
Married	11,175 (82.4)	2,380 (17.6)	​	22,510 (97.1)	663 (2.9)	​
Divorced/Widowed	641 (78.3)	178 (21.7)	​	1,609 (96.6)	56 (3.4)	​
Unmarried	65 (62.5)	39 (37.5)	​	173 (96.1)	7 (3.9)	​
Education, n (%)	​	​	**<0.001**	​	​	**0.002**
Undergraduate/graduate	580 (89.2)	70 (10.8)	​	747 (98.8)	9 (1.2)	​
High school/technical school	1741 (84.6)	317 (15.4)	​	2,552 (97.2)	73 (2.8)	​
Primary/middle school	7,879 (82.4)	1,686 (17.6)	​	17,146 (97.3)	479 (2.7)	​
Illiterate/semi-literate	1,681 (76.2)	524 (23.8)	​	3,847 (95.9)	165 (4.1)	​
Hypertension history, n (%)	​	​	**<0.001**	​	​	**<0.001**
Yes	1,589 (72.1)	615 (27.9)	​	4,686 (95.9)	198 (4.1)	​
No	10,292 (83.9)	1982 (16.1)	​	19,606 (97.4)	528 (2.6)	​
Smoking status, n (%)	​	​	**<0.001**	​	​	**<0.001**
Ever/Current	2,979 (75.6)	961 (24.4)	​	6,471 (95.8)	284 (4.2)	​
Never	8,223 (84.9)	1,459 (15.1)	​	17,814 (97.6)	441 (2.4)	​
Alcohol drinking, n (%)	​	​	**<0.001**	​	​	**<0.001**
Ever/Current	2,499 (77.0)	746 (23.0)	​	5,410 (96.3)	209 (3.7)	​
Never	9,332 (83.8)	1808 (16.2)	​	18,875 (97.3)	516 (2.7)	​
Tea drinking, n (%)	​	​	**<0.001**	​	​	**<0.001**
Ever/Current	2,488 (79.6)	636 (20.4)	​	4,417 (96.1)	177 (3.9)	​
Never	9,338 (83.0)	1916 (17.0)	​	19,661 (97.3)	538 (2.7)	​
Physical activity, n (%)	​	​	**0.001**	​	​	0.178
Moderate/high	957 (78.8)	258 (21.2)	​	1,067 (97.3)	30 (2.7)	​
Low	10,824 (82.6)	2,285 (17.4)	​	22,741 (97.9)	495 (2.1)	​
Daily vegetable intake, n (%)	​	​	**<0.001**	​	​	**<0.001**
≤250 g/day	6,772 (80.9)	1,602 (19.1)	​	17,435 (96.7)	586 (3.3)	​
>250 g/day	5,034 (84.2)	946 (15.8)	​	6,834 (98.0)	139 (2.0)	​

Baseline characteristics are presented as mean ± standard deviation for approximately normally distributed continuous variables, or as median (interquartile range) for non-normally distributed variables, and as n (%) for categorical variables. Percentages in categorical rows indicate row percentages by incident major NCD status. Group differences were assessed using Welch’s t test or the Wilcoxon rank-sum test for continuous variables, and the χ^2^ test or Fisher’s exact test for categorical variables, as appropriate. Missing categories are not separately shown in this table and are summarized in Supplementary Table S3; P values for categorical variables were calculated using non-missing categories. A two-sided P value <0.05 was considered statistically significant. Bold values indicate statistically significant P values (P < 0.05). Abbreviations: NCDs, non-communicable diseases.

The missingness of blood biomarkers in both cohorts is presented in Supplementary Table S1. As an overall health endpoint, we first examined associations between baseline blood biomarkers and all-cause mortality in the discovery cohort (Supplementary Figure S3). Tumor markers, inflammation-related markers, and markers of liver/kidney function and nutritional status were all significantly associated with mortality risk, suggesting that routine examination biomarkers capture long-term health risk across multiple physiological domains.

**FIGURE 1:**
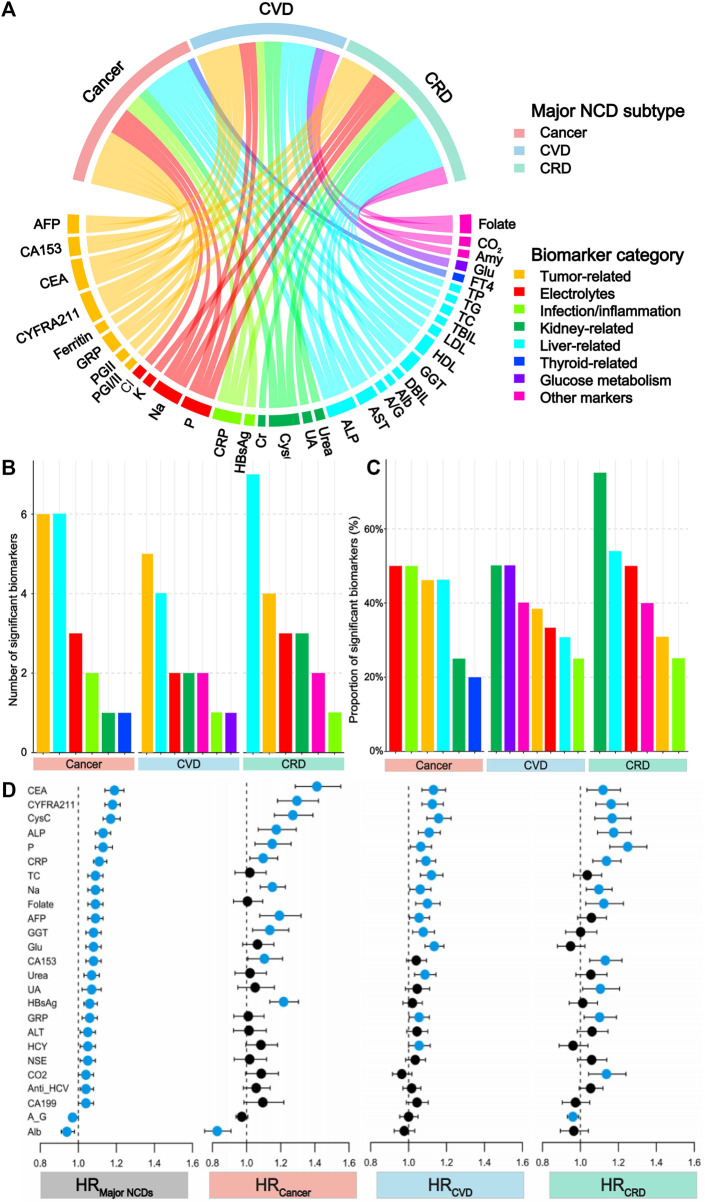
Associations between biomarkers and incidence of major non-communicable diseases (Taizhou Longitudinal Study, Taizhou, China, 2011–2023). **(A)** Circos plot of significant biomarker associations across major NCD subtypes. **(B,C)** Number and proportion of significant biomarkers by category and disease subtype. **(D)** Forest plots of HRs and 95% CIs per 1-standard-deviation increase in baseline biomarkers; blue and black symbols indicate significant and non-significant associations, respectively. Biomarker abbreviations are defined in Supplementary Table S1. Abbreviations: CI, confidence interval; CRD, chronic respiratory diseases; CVD, cardio-cerebrovascular diseases; HR, hazard ratio; NCDs, non-communicable diseases.

We then assessed associations between blood biomarkers and incident major NCDs and their subtypes (Figure 1). Figures 1A–C summarize significant biomarker associations across major NCD subtypes using a Circos plot and category-level counts and proportions. For cancer, tumor markers and liver function markers contributed the largest absolute counts, while several electrolytes, including chloride, sodium, and phosphate, were prominent in relative abundance. For CVD and CRD, kidney function markers such as cystatin C and creatinine showed strong overlap, whereas glucose showed a more CVD-specific pattern. Figure 1D shows adjusted Cox proportional hazards results for incident major NCDs and subtype outcomes. Overall, 25 circulating blood biomarkers were significantly associated with incident major NCDs, including 23 positively associated and 2 inversely associated biomarkers.

### Construction of the NIMO score

After applying the prespecified Cox pre-screening and three-method consensus selection framework in the discovery cohort, 19 predictors were retained for construction of the NIMO score. The final NIMO score included 15 circulating blood biomarkers, plus age, smoking status, hypertension, and daily vegetable intake. Their relevance to major NCD risk is summarized in Supplementary Table S4. Supplementary Figure S4 shows selected predictors, cut-offs, and coefficient-based point assignments used to construct the NIMO score in the discovery cohort. Participants were then classified into low-, intermediate-, and high-risk groups according to the final score.

### Performance of the NIMO score in the discovery cohort

In the discovery cohort, the NIMO score showed clear risk gradients for incident major NCDs (Supplementary Figure S5). The high-risk group represented 25.0% of participants but accounted for 44.7% (1162/2597) of incident major NCD events, with a 4.75-fold higher risk than the low-risk group (95% CI: 4.17–5.41). The NIMO score showed moderate discrimination for incident major NCDs, with an AUC of 0.70.

Similar gradients were observed for all-cause mortality. The high-risk group accounted for 55.9% (353/632) of deaths and had a 7.56-fold higher mortality risk than the low-risk group (95% CI: 5.47–10.46). The AUC for all-cause mortality was 0.73.

### Temporal validation and robustness analyses

**FIGURE 2:**
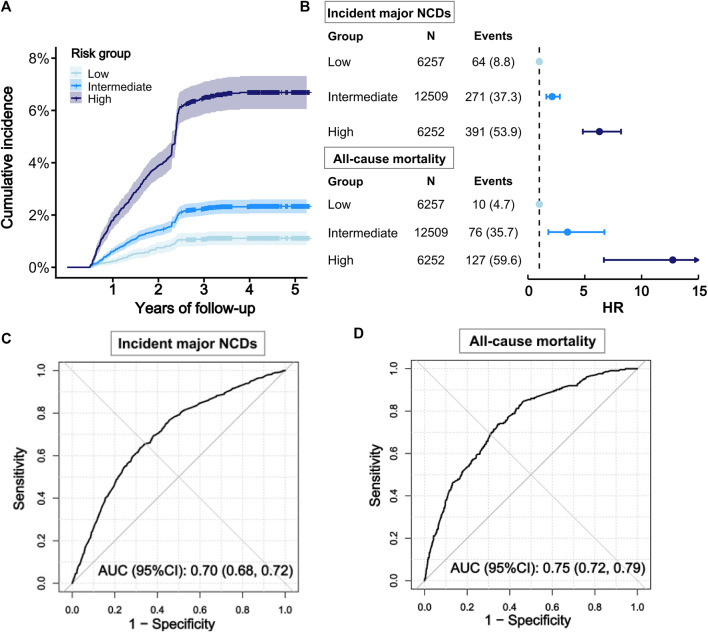
Risk stratification performance of the main score in the temporal validation cohort (Taizhou Longitudinal Study, Taizhou, China, 2011–2023). **(A)** Cumulative incidence of incident major NCDs by risk group. **(B)** HRs and 95% CIs for incident major NCDs and all-cause mortality by risk group. Percentages indicate the proportion of all events captured by each risk group. **(C,D)** Receiver operating characteristic curves for incident major NCDs and all-cause mortality. The main score refers to the NIMO score. Abbreviations: AUC, area under the receiver operating characteristic curve; CI, confidence interval; HR, hazard ratio; NCDs, non-communicable diseases; NIMO, Major NCDs Identification Model.

In the temporal validation cohort, the NIMO score retained clear risk stratification for incident major NCDs (Figure 2). The high-risk group represented 25.0% of participants but accounted for 53.9% (391/726) of incident major NCD events, with a 6.29-fold higher risk than the low-risk group (95% CI: 4.83–8.19). Similar gradients were observed for all-cause mortality: the high-risk group accounted for 59.6% (127/213) of deaths and had a 12.74-fold higher mortality risk than the low-risk group. The NIMO score showed moderate discrimination in the temporal validation cohort, with both AUCs and C-indexes of 0.70 for incident major NCDs and 0.75 for all-cause mortality.

Sensitivity analyses supported the robustness of the NIMO score, with generally consistent risk gradients across subgroup, missing-data, risk-threshold, predictor-selection, continuous-biomarker, expanded-outcome, glucose-excluded, and fixed 3-year analyses (Supplementary Figure S6; Supplementary Tables S5, S6). The composite-outcome findings were not solely driven by CVD: cancer and CRD together accounted for 42.0% and 39.8% of first events within the original composite outcome in the discovery and temporal validation cohorts, respectively, and the score retained stratification for non-CVD outcomes and for cancer, CVD, and CRD separately (Supplementary Table S7). Repeated 7:3 internal validation showed stable discrimination and risk gradients, and bootstrap analyses showed all-three consensus frequencies of 80%–100% for the selected predictors (Supplementary Table S8).

### Comparison with disease-specific scores

**FIGURE 3:**
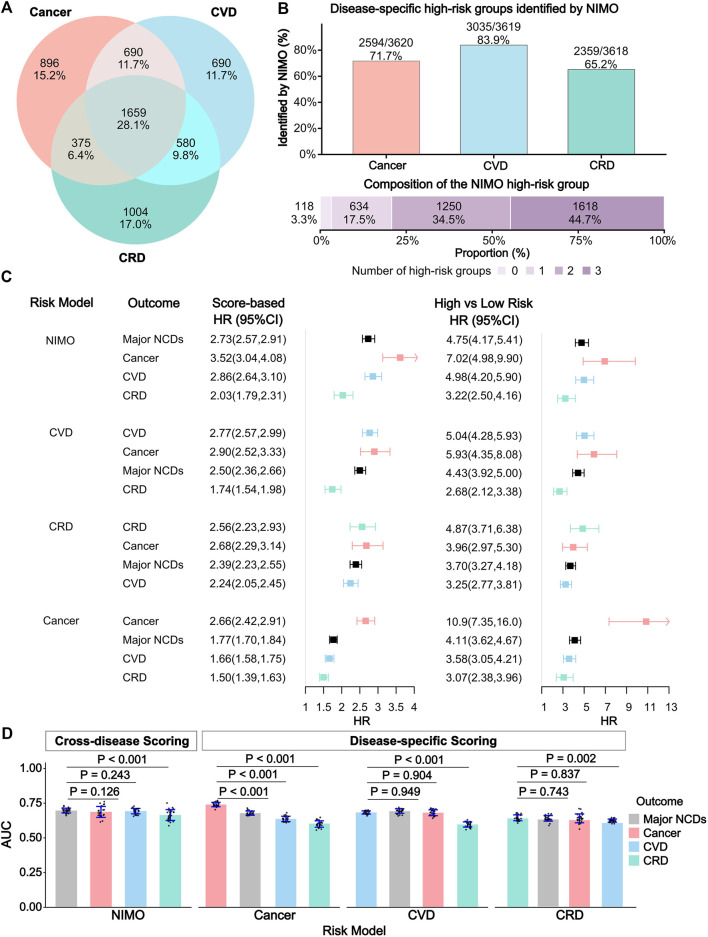
Comparison of the main score and disease-specific scores for major non-communicable diseases (Taizhou Longitudinal Study, Taizhou, China, 2011–2023). **(A)** Venn diagram illustrating the overlap of high-risk groups identified by cancer-, CVD-, and CRD-specific scores. Numbers and percentages denote the size and proportion of overlapping populations. **(B)** Overlap between the main-score high-risk group and disease-specific high-risk groups. **(C,D)** HR and AUC comparisons for the main score and disease-specific scores across major NCDs, cancer, CVD, and CRD. The main score refers to the NIMO score. Abbreviations: AUC, area under the receiver operating characteristic curve; CI, confidence interval; CRD, chronic respiratory diseases; CVD, cardio-cerebrovascular diseases; HR, hazard ratio; NCDs, non-communicable diseases; NIMO, Major NCDs Identification Model.

As disease-focused comparators, the cancer-, CVD-, and CRD-specific scores were compared with the NIMO score. The selected predictors for the main and disease-specific scores are summarized in Supplementary Table S9. The four scores shared overlapping predictors and high-risk population characteristics. Shared predictors included age, smoking status, daily vegetable intake, and several circulating blood biomarkers, such as carcinoembryonic antigen, cytokeratin 19 fragment, C-reactive protein, and cystatin C. Additionally, Figure 3A showed that among individuals identified as high risk by disease-specific scores, 56.1% were classified as high risk by at least two disease-specific scores, and 28.1% were classified as high risk by all three. Figure 3B further showed that the NIMO high-risk group captured 71.7%, 83.9%, and 65.2% of cancer-, CVD-, and CRD-specific high-risk individuals, respectively. Within the NIMO high-risk group, 79.2% also met high-risk criteria for at least two disease-specific scores.

In risk-stratification analyses, the NIMO score showed clear high-versus-low risk gradients across the composite and subtype outcomes, with HRs of 4.75 for major NCDs, 7.02 for cancer, 4.98 for CVD, and 3.22 for CRD (Figure 3C). Disease-specific scores generally showed stronger gradients for their target outcomes, but several non-target outcomes also showed elevated risks, suggesting cross-disease risk clustering. In discrimination analyses, disease-specific scores tended to achieve higher AUCs for their target outcomes, whereas the NIMO score showed relatively stable discrimination across the composite and subtype outcomes (Figure 3D). Together, these findings suggest that disease-specific scores may be more suitable for focused single-disease risk assessment, whereas the NIMO score provides a first-step integrated stratification approach for identifying individuals with elevated overall major NCD risk before further disease-specific evaluation.

### Comparison with traditional-factor and blood-based scores

As information-source comparators, the traditional-factor and blood-based scores were compared with the NIMO score. The selected predictors are summarized in Supplementary Table S9. In both cohorts, all three scores showed graded risk stratification for incident major NCDs and all-cause mortality (Supplementary Table S10). The blood-based and traditional-factor scores showed broadly comparable performance, suggesting that both information sources contributed to risk stratification. Discrimination analyses showed that the NIMO score had slightly higher AUCs than either comparator score, whereas the blood-based and traditional-factor scores did not differ significantly (Supplementary Figure S7). These findings suggest that integrating circulating blood biomarkers with traditional factors provided more stable risk stratification and modestly improved discrimination.

## Discussion

Blood tests are among the most common health examination items, but current reports mainly show whether individual biomarker is within the reference range, lacking an integrated interpretation of future health risks. As an overall health endpoint, all-cause mortality reflects long-term health outcomes independent of specific disease diagnoses. Previous studies have linked combinations of biomarkers to mortality risk [10, 20]. Extending this evidence, our prospective analysis shows that baseline blood biomarker profiles are significantly associated with subsequent all-cause mortality, suggesting that blood profiles may serve as early indicators of overall health status. For context, previous biomarker-based models for all-cause mortality reported C-statistics of approximately 0.66–0.79 [10, 21–23]. Although NIMO was developed primarily for incident major NCD risk stratification, it showed a comparable C-index of 0.75 for all-cause mortality in the temporal validation cohort, suggesting potential value for overall risk identification in health examination settings.

Blood biomarkers showed both shared and disease-specific association patterns across cancer, CVD, and CRD. Tumor markers, electrolytes, and kidney- and liver-function related markers appeared across all three outcomes, whereas some biomarkers showed more disease-specific patterns. Together with prior evidence that cancer, CVD, and CRD share key risk factors such as smoking, obesity, and inflammation [11, 24–26], these findings suggest that major NCD subtypes may have partially shared risk bases. Consistently, the disease-specific scores also shared several selected predictors across cancer, CVD, and CRD, supporting the interpretation that the main score captured partly shared multisystem risk signals. These biomarker associations should be interpreted as risk-stratification signals rather than disease-specific diagnostic indicators or direct mechanistic evidence. For example, associations between tumor-related markers and non-cancer outcomes may reflect broader subclinical disease burden, inflammation, nutritional status, or multisystem health status [27]. In practice, the NIMO score is intended to help prioritize individuals for further health management, follow-up, lifestyle intervention, or targeted assessment, rather than to diagnose a specific disease based on any single biomarker.

In this study, we developed and temporally validated NIMO, a multi-outcome risk stratification model designed for health examination settings to support major NCD risk stratification. Using a supervised multi-method variable-selection framework, NIMO integrates epidemiological, clinical, and biomarker information and provides a practical framework based on information that can be obtained in comprehensive health examination settings. The NIMO score showed stable risk stratification for incident major NCDs. In the temporal validation cohort, the high-risk group accounted for more than half of incident major NCD events and had an approximately sixfold higher risk than the low-risk group. The score also showed clear risk gradients for cancer, CVD, and CRD. As a secondary outcome, all-cause mortality showed a similar gradient, with high-risk versus low-risk HRs of 7.56 in the discovery cohort and 12.74 in the temporal validation cohort, suggesting that the score also captured broader overall health risk.

The disease-specific scores also revealed substantial overlap in high-risk populations, with 56.1% of disease-specific high-risk individuals meeting high-risk criteria for at least two disease-specific scores. In the temporal validation cohort, the NIMO high-risk group represented 25.0% of participants but accounted for 53.9% of incident major NCD events. This finding indicates that blood profile–based joint assessment may substantially improve the efficiency of early identification of high-risk populations. Although previous studies on joint modeling of multiple chronic diseases are limited and concerns have been raised about performance loss in multi-outcome settings [28], our results show that NIMO maintains stable stratification across heterogeneous outcomes, supporting its feasibility for integrated risk assessment. From a public health practice perspective, disease-specific screening strategies not only require multiple independent assessments in time and cost, but may also lead individuals to overly focus on known risks, weakening active attention to other potential NCD risks. In contrast, multi-disease risk stratification can reveal multiple risk levels simultaneously, helping individuals more comprehensively understand their health status and reduce cognitive limitations from single risk information. Additionally, the broadly comparable performance of the blood-based and traditional-factor scores suggests that both information sources contributed to risk stratification. Their integration in the NIMO score provided more stable stratification and modestly improved discrimination.

This study has several key strengths. First, it used a large community-based prospective cohort with a later-enrolled temporal validation cohort, reducing selection and recall biases and supporting temporal reproducibility. Second, it included 54 circulating blood biomarkers across multiple systems, including tumor markers, glycometabolic markers, electrolytes, infection markers, and thyroid-, kidney-, and liver-function related markers, together with questionnaire- and physical examination-derived indicators. By additionally examining all-cause mortality as a secondary outcome, this study strengthened evidence that blood profile-based stratification reflects broader overall health risk. Finally, multiple robustness analyses, including alternative missing-data approaches, risk-group thresholds, predictor-selection strategies, continuous biomarker modeling, expanded outcome definitions, repeated 7:3 internal validation, and bootstrap variable-selection stability, supported the main findings.

### Limitations

Several limitations must be acknowledged. First, blood samples were stored for varying durations before centralized testing, and some degradation might have occurred despite standard protocols. However, reliability tests of key biomarkers showed high correlations (63%–94%). Second, all-cause mortality was used only as a secondary outcome, and the temporal validation cohort had relatively short follow-up with limited death events. Third, diabetes was not included in the primary composite outcome because of its close relationship with baseline glucose. Although expanded-outcome and glucose-excluded analyses partly addressed this issue, they cannot replace dedicated modeling of diabetes and its complications. Fourth, the cut-off-based scoring approach improved interpretability but may have caused information loss and may depend on cohort-specific distributions and outcome patterns [29–32]; therefore, the stability of cut-offs and point assignments requires further external validation. Single imputation may also underestimate uncertainty, although continuous biomarker modeling, multiple-imputation, and complete-case analyses showed consistent results. Finally, some biomarkers, particularly tumor-related markers, may not be routinely measured in all health examination settings, and implementation should consider test availability, cost, and laboratory platform consistency. Participants were recruited from a single geographical area, and further external validation in different regions and populations is needed.

### Conclusion

This study developed and temporally validated the NIMO model using a large population-based cohort, proposing a blood biomarker–based framework for major NCD risk identification. The model integrates blood biomarkers with basic epidemiological information, reflecting multisystem health status and identifying shared risk directions for major NCDs from overall blood profiles. In the temporal validation cohort, the high-risk group accounted for more than half of incident major NCD events and had an approximately sixfold higher risk than the low-risk group, while also showing substantially elevated all-cause mortality risk. Furthermore, comparisons with disease-specific scores further supported NIMO’s potential value for both composite and subtype risk stratification.
