# Supplementary material for: Multimodal risk profiles reveal shared and disease-specific risks of major non-communicable diseases: a prospective cohort study of 42,666 individuals
Source: Int J Public Health. 2026 Jul 9;71:1609591. doi: 10.3389/ijph.2026.1609591 (PMC13391432; doi:10.3389/ijph.2026.1609591)
Supplement: Supplementary file 1 [file DataSheet1.pdf]

International Journal of Public Health
Supplementary Material
Multimodal Risk Profiles Reveal Shared and Disease-Specific Risks of Major Non-Communicable Diseases: A Prospective Cohort Study of 42,666 Individuals

Figure S1: Study population selection and overall analytic workflow (Taizhou, China, 2011–2023)

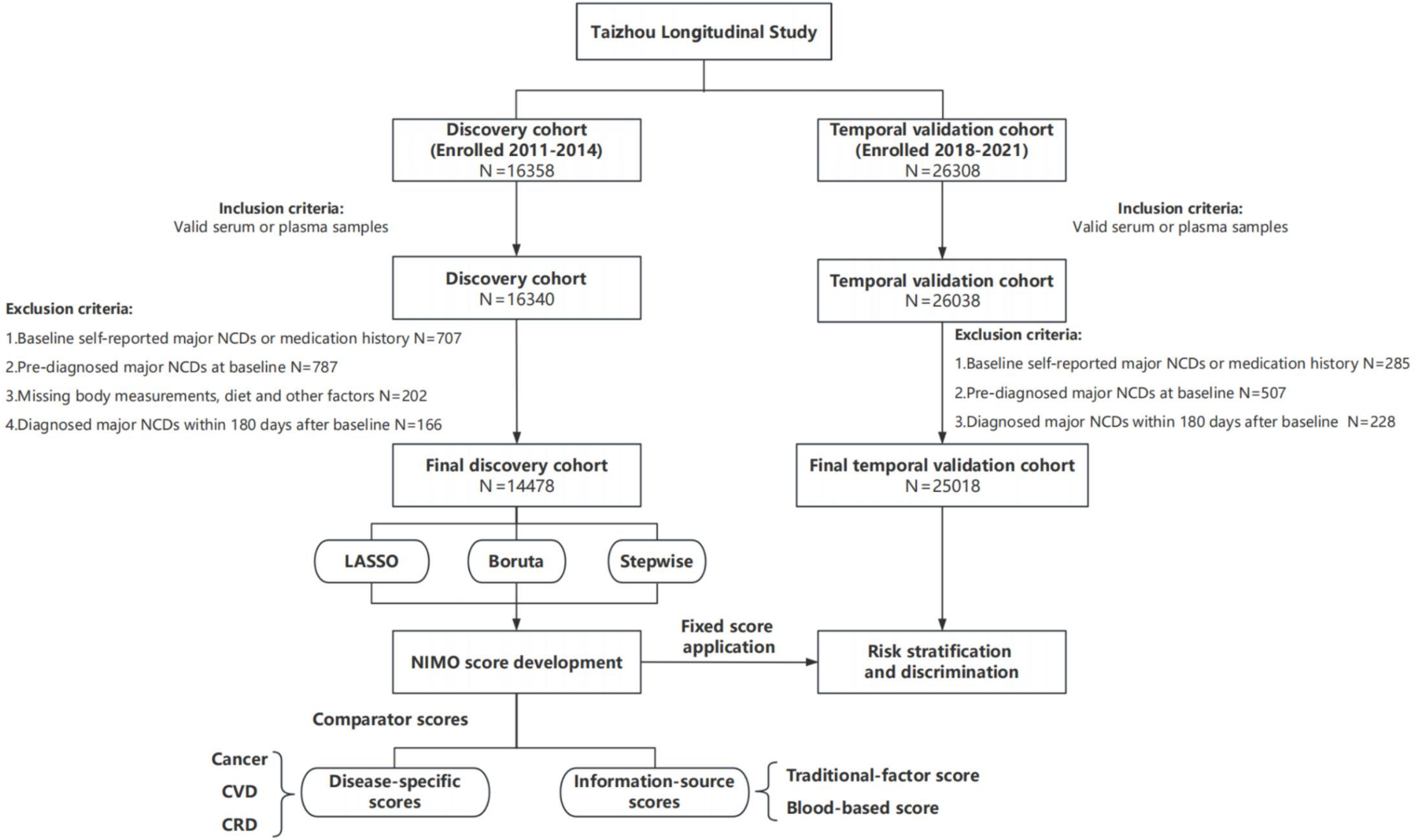

Legend: Abbreviations: LASSO, least absolute shrinkage and selection operator; Boruta, Boruta random forest feature selection; Stepwise, stepwise regression; NCDs, non-communicable diseases; CVD, cardio-cerebrovascular diseases; CRD, chronic respiratory diseases.

Table S1: Circulating blood biomarkers and missing rates in the discovery and temporal validation cohorts (Taizhou, China, 2011–2021)

Circulating blood biomarkers	Abbreviation	Unit	Missing Rate %		Circulating blood biomarkers	Abbreviation	Unit	Missing Rate %	
			Discovery	Validation				Discovery	Validation
**Tumor-related biomarkers**					Creatinine	Cr	μmol/L	0.4	0.3
Alpha-Fetoprotein	AFP	ng/mL	1.5	0.7	Cystatin C	CysC	mg/L	0.2	0.2
Carbohydrate Antigen 125	CA125	U/mL	0.9	0.4	Estimated Glomerular Filtration Rate	eGFR	ml/min	0.4	0.3
Carbohydrate Antigen 15-3	CA153	U/mL	1	0.3	**Electrolytes**				
Carbohydrate Antigen 19-9	CA199	U/mL	1.3	0.4	Calcium	Ca	mmol/L	0.4	0.6
Carcinoembryonic Antigen	CEA	ng/mL	1.1	0.2	Chloride	Cl	mmol/L	0.1	0.1
Cytokeratin 19 Fragment	CYFRA21-1	ng/mL	1.3	0.5	Potassium	K	mmol/L	0.1	0.1
Ferritin	Ferritin	ng/mL	0.9	0.3	Magnesium	Mg	mmol/L	0.4	0.2
Pro-Gastrin-Releasing Peptide	GRP	pg/mL	1.1	0.3	Sodium	Na	mmol/L	0.2	0.6
Neuron-Specific Enolase	NSE	ng/mL	0.9	0.4	Phosphorus	P	mmol/L	0.4	0.2
Pepsinogen I	PGI	ng/ml	2.1	1	**Thyroid-related biomarkers**				
Pepsinogen I/II Ratio	PGI/II	/	4.7	2.5	Free Triiodothyronine	FT3	pmol/L	0.7	0.2
Pepsinogen II	PGII	ng/ml	2.8	1.1	Free Thyroxine	FT4	pmol/L	1.4	0.2
Squamous Cell Carcinoma Antigen	SCCAg	ng/mL	1.6	0.4	Triiodothyronine	T3	nmol/L	0.9	0.2
**Liver-related biomarkers**					Thyroxine	T4	nmol/L	1.4	0.2
A/G Ratio	A/G	/	0.4	0.2	Thyroid-Stimulating Hormone	TSH	mIU/L	0.8	0.2
Albumin	Alb	g/L	0.4	0.2	**Infection/inflammation**				
Alkaline Phosphatase	ALP	U/L	0.4	0.2	Hepatitis C Virus Antibody	Anti-HCV	C.O.I.	2.3	0.7
Alanine Aminotransferase	ALT	U/L	0.4	0.1	C-reactive Protein	CRP	mg/L	0.3	0.3
Aspartate Aminotransferase	AST	U/L	0.6	0.3	Hepatitis B Surface Antigen	HBsAg	IU/mL	1.8	0.8
Direct Bilirubin	DBIL	μmol/L	0.4	0.2	Helicobacter Pylori Antibody	HpAb	/	0.1	1.6
γ-Glutamyltransferase	GGT	U/L	0.4	0.2	Glucose metabolism				
Globulin	Glob	g/L	0.4	0.2	Glucose	Glu	mmol/L	0.4	0.3
High-Density Lipoprotein	HDL	mmol/L	0.4	0.1	Insulin	Insulin	pmol/l	1.5	0.7
Indirect Bilirubin	IBIL	μmol/L	0.6	0.9	**Other biomarkers**				
Low-Density Lipoprotein	LDL	mmol/L	0.4	0.3	Amylase	Amy	U/L	0.5	0.6
Total Bilirubin	TBIL	μmol/L	0.4	0.6	Carbon Dioxide	CO_2	mmol/L	0.4	0.6
Total Cholesterol	TC	mmol/L	0.4	0.2	Folate	Folate	nmol/L	2.5	0.6
Triglyceride	TG	mmol/L	0.4	0.3	Homocysteine	HCY	μmol/L	0	0.2
Total Protein	TP	g/L	0.4	0.2	Uric Acid	UA	μmol/L	0.4	0.6
**Kidney-related biomarkers**					Urea	Urea	mmol/L	0.3	0.2

Note: Missing rates are presented as percentages. Discovery refers to the discovery cohort, and Validation refers to the temporal validation cohort.

Table S2: Candidate variable categories and information represented in relation to major non-communicable disease risk (Taizhou, China, 2011–2021)

Variable category	Information represented and relevance to major non-communicable disease risk
Tumor-related biomarkers	Tumor-related, gastrointestinal-related, and iron metabolism-related indicators, reflecting potential subclinical disease burden and overall health status.
Liver-related biomarkers	Liver function, bilirubin metabolism, protein/nutritional status, and lipid metabolism, reflecting metabolic status and organ function.
Kidney-related biomarkers	Kidney function-related information, reflecting organ function, metabolic burden, and overall health risk.
Electrolytes	Electrolyte balance and internal homeostasis, reflecting renal regulation, mineral metabolism, and cardiovascular function.
Thyroid-related biomarkers	Thyroid function-related information, reflecting endocrine function, basal metabolism, and metabolic homeostasis.
Infection/inflammation biomarkers	Inflammatory burden and common infection-related status, reflecting immune-inflammatory background and potential chronic disease risk.
Glucose metabolism biomarkers	Glucose- and insulin-related metabolic function, reflecting baseline metabolic risk.
Other biomarkers	Additional information on overall metabolic homeostasis, nutritional status, and related organ function.
Demographic and socioeconomic factors	Basic demographic and socioeconomic background, including age, sex, ethnicity, education, marital status, and family income, which may be related to baseline major NCD risk and differences in health-related resources.
Lifestyle factors	Common modifiable behavioral factors, including smoking, alcohol drinking, tea drinking, and physical activity, which are closely related to major NCD risk.
Anthropometric and body composition factors	Anthropometric and body composition information, including body mass index, body fat percentage, height, and weight, reflecting obesity-related and metabolism-related risk.
Blood pressure-related factors	Hypertension history and measured blood pressure indicators, reflecting cardiovascular and metabolic risk burden.
Dietary factors	Major dietary intake information, including vegetables, fruits, meat, dairy products, soy products, fried foods, cured meat, pickled vegetables, and smoked foods, reflecting diet-related chronic disease risk.

Table S3: Definitions and screening status of questionnaire and physical examination variables (Taizhou, China, 2011–2021)

Variable	Coding / unit	Missing Rate %		Status
		Discovery cohort	Temporal validation cohort	
**Demographic/socioeconomic**				
Age	years	0.00	0.00	Retained
Sex	Male; female	0.00	0.00	Retained
Ethnicity	Han; other ethnic groups	0.00	0.31	Cox-filtered
Education	Undergraduate/graduate; high school/technical school; middle/primary school; illiterate/semi-literate	0.00	0.00	Retained
Marital status	Married; divorced; widowed; unmarried	0.00	0.00	Retained
Family income	<10,000 RMB; 10,000–20,000 RMB; 20,000–35,000 RMB; ≥35,000 RMB	2.35	36.34	Missingness
**Lifestyle factors**				
Smoking status	Never; ever; current	5.91	0.03	Retained
Alcohol drinking	Never; ever; current	0.64	0.03	Retained
Tea drinking	Never; ever; current	0.69	0.90	Retained
Physical activity	Low; moderate; high	1.06	2.74	Retained
**Anthropometrics**				
Body mass index	kg/m^2	0.20	0.05	Retained
Body fat percentage	%	4.83	1.05	Cox-filtered
Height	cm	0.25	0.05	Overlap
Weight	kg	0.26	0.05	Overlap
**Blood pressure-related**				
Hypertension history	No; yes	0.00	0.00	Retained
Systolic blood pressure	mmHg	0.55	0.78	Overlap
Diastolic blood pressure	mmHg	0.55	0.78	Overlap
**Dietary factors**				
Daily vegetable intake	g/day	0.86	0.10	Retained
Fruit intake	g/day	29.22	20.06	Missingness
Fried food intake	g/day	67.47	71.78	Missingness
Dairy intake	g/day	54.69	44.75	Missingness
Soy product intake	g/day	5.30	7.93	Cox-filtered
Meat intake	g/day	2.51	2.29	Cox-filtered
Cured meat intake	g/day	68.23	73.68	Missingness
Pickled vegetable intake	g/day	27.66	35.03	Missingness
Smoked food intake	g/day	89.72	89.05	Missingness

Note: Status: Retained, retained for subsequent candidate screening; Missingness, excluded because missingness exceeded 20% in either cohort; Overlap, not entered separately because its information overlapped with a derived or clinically representative variable; Cox-filtered, not retained after the prespecified Cox pre-screening step.

Figure S2: Predictor selection and risk score construction workflow (Taizhou, China, 2011–2023)

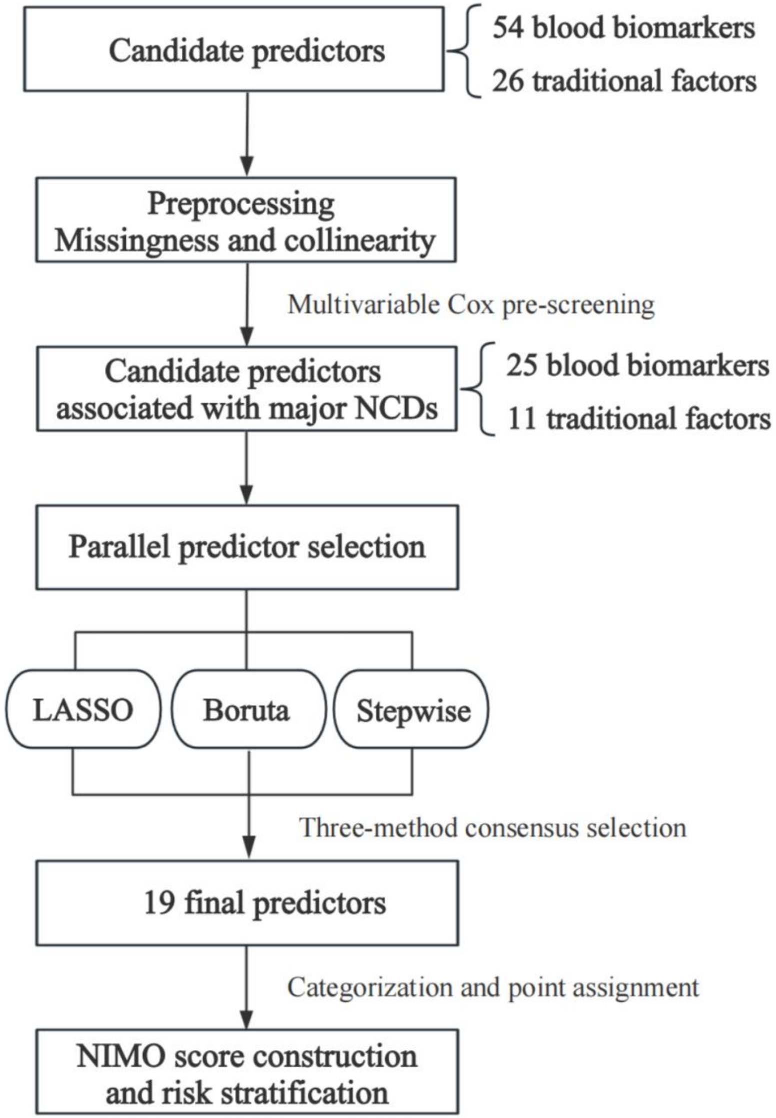

Legend: Abbreviations: LASSO, least absolute shrinkage and selection operator; Boruta, Boruta random forest feature selection; Stepwise, stepwise regression; NIMO, Major NCDs Identification Model; NCDs, non-communicable diseases

Figure S3: Associations between baseline blood biomarkers and all-cause mortality in the discovery cohort (Taizhou, China, 2011–2023)

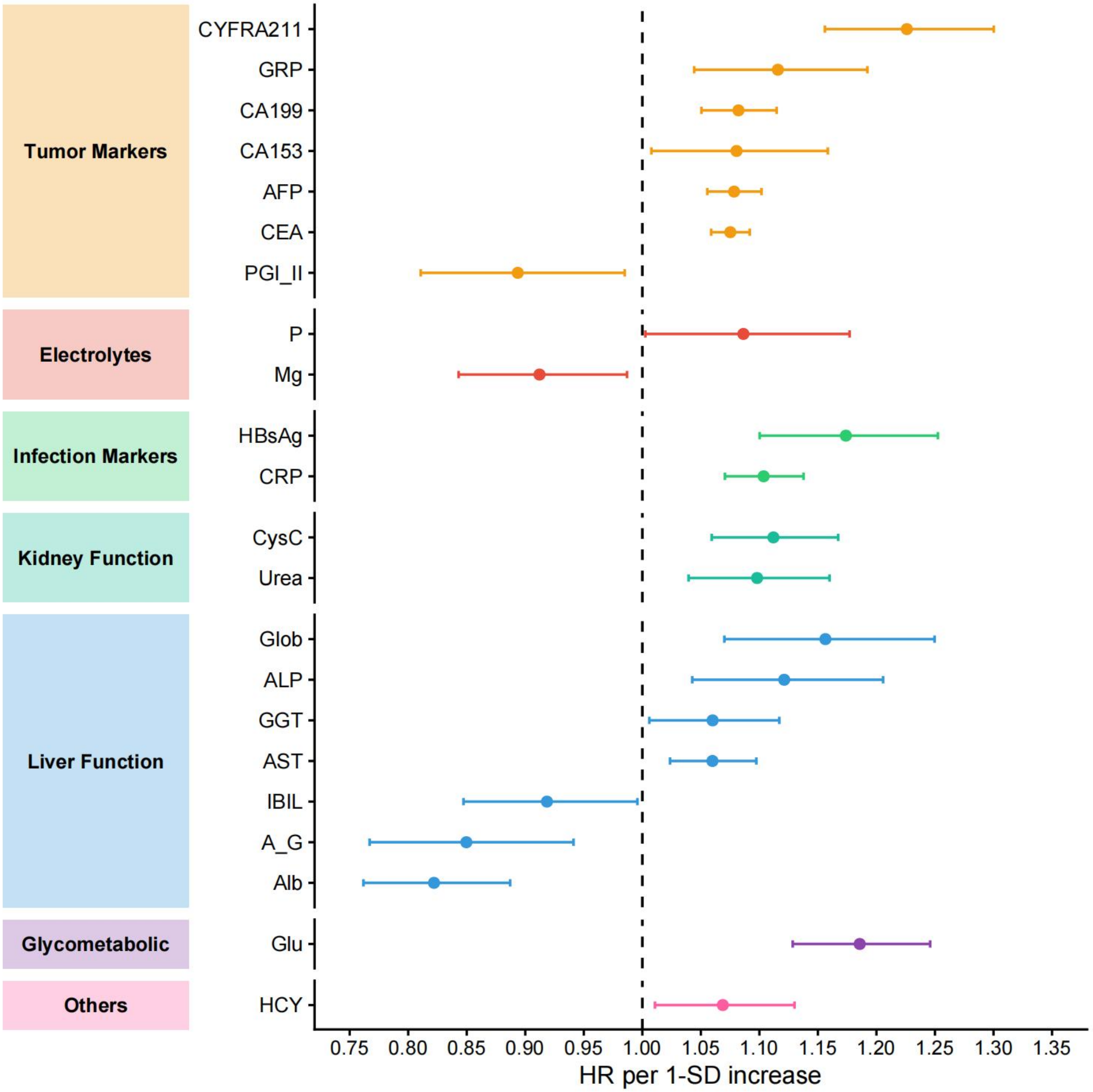

Legend: Hazard ratios and 95% confidence intervals for all-cause mortality were estimated per 1-standard-deviation increase in baseline circulating blood biomarkers. Estimates were obtained from multivariable Cox proportional hazards models adjusted for age, sex, body mass index, smoking status, alcohol drinking, hypertension, and daily vegetable intake. Biomarker abbreviations are defined in Table S1.

Table S4: Evidence-based relevance of predictors included in the main score (Taizhou, China, 2011–2023)

Predictor	Domain	Relevance to major NCD risk	Evidence
Age	Demographic	A core demographic factor in the development of multiple chronic diseases.	S1, S2
Smoking status	Lifestyle	An important modifiable risk factor for cancer, cardio-cerebrovascular diseases, chronic respiratory diseases, and mortality.	S3
Hypertension history	Blood pressure -related	A core risk factor for cardio-cerebrovascular diseases, and also related to overall chronic disease burden and some cancer risks.	S4, S5
Daily vegetable intake	Dietary	Reflects diet quality and has been associated with cardiovascular disease, total cancer, and mortality risks.	S6
AFP	Tumor-related	A serum marker related to hepatocellular carcinoma, and may also reflect liver-related disease burden and overall health-risk signals.	S7, S8
CA153	Tumor-related	A serum marker related to breast cancer, and may also reflect cardiovascular events and mortality risk in the general population.	S7, S9
CEA	Tumor-related	A commonly used tumor marker associated with gastrointestinal tumors, pulmonary disease, and cardiovascular/mortality risk.	S7, S10, S11
CYFRA21-1	Tumor-related	A lung cancer-related marker, also associated with chronic respiratory disease phenotypes and overall health-risk signals.	S7, S12, S13
HBsAg	Infection/ inflammation	Reflects hepatitis B virus infection and is related to infection-attributable cancers such as hepatocellular carcinoma.	S14, S15
Anti-HCV	Infection/ inflammation	Reflects hepatitis C virus infection and is related to infection-attributable cancers and atherosclerotic cardiovascular risk.	S15, S16
CRP	Infection/ inflammation	Reflects systemic inflammation and is associated with coronary heart disease, stroke, mortality, and multiple cancer risks.	S17, S18
Alb	Liver-related	Reflects nutritional, liver-function, and inflammation-related health status, and is associated with cancer and chronic respiratory disease mortality risks.	S19, S20
ALP	Liver-related	Reflects hepatobiliary and bone-mineral metabolism status, and is associated with cardiovascular disease, mortality, and cancer risk.	S21, S22
TC	Liver-related	Reflects lipid metabolism status and is commonly used in cardiovascular disease risk assessment.	S23
CysC	Kidney-related	Reflects kidney function and systemic health status, and is associated with cardiovascular mortality, all-cause mortality, and chronic respiratory diseases.	S24, S25
Glu	Metabolic/other	Reflects glucose metabolism status and is associated with vascular disease, mortality, and some cancer-related burden.	S26, S27
Folate	Metabolic/other	Reflects nutritional and metabolic status, and is associated with colorectal cancer and mortality risks.	S28, S29
Na	Electrolytes	Reflects fluid-electrolyte balance and internal homeostasis, and is associated with chronic disease development, aging, and mortality risk.	S30
P	Electrolytes	Reflects mineral metabolism and internal homeostasis, and is associated with cardiovascular disease and chronic respiratory disease mortality.	S31, S32

Note: Evidence summarizes selected literature supporting the relevance of the predictors included in the main score to major non-communicable disease risk. The main score refers to the NIMO score. Abbreviation: NCD, non-communicable disease; NIMO, Major NCDs Identification Model.

Figure S4: Selected predictors, cut-offs, and point assignments of the main score (Taizhou, China, 2011–2023)

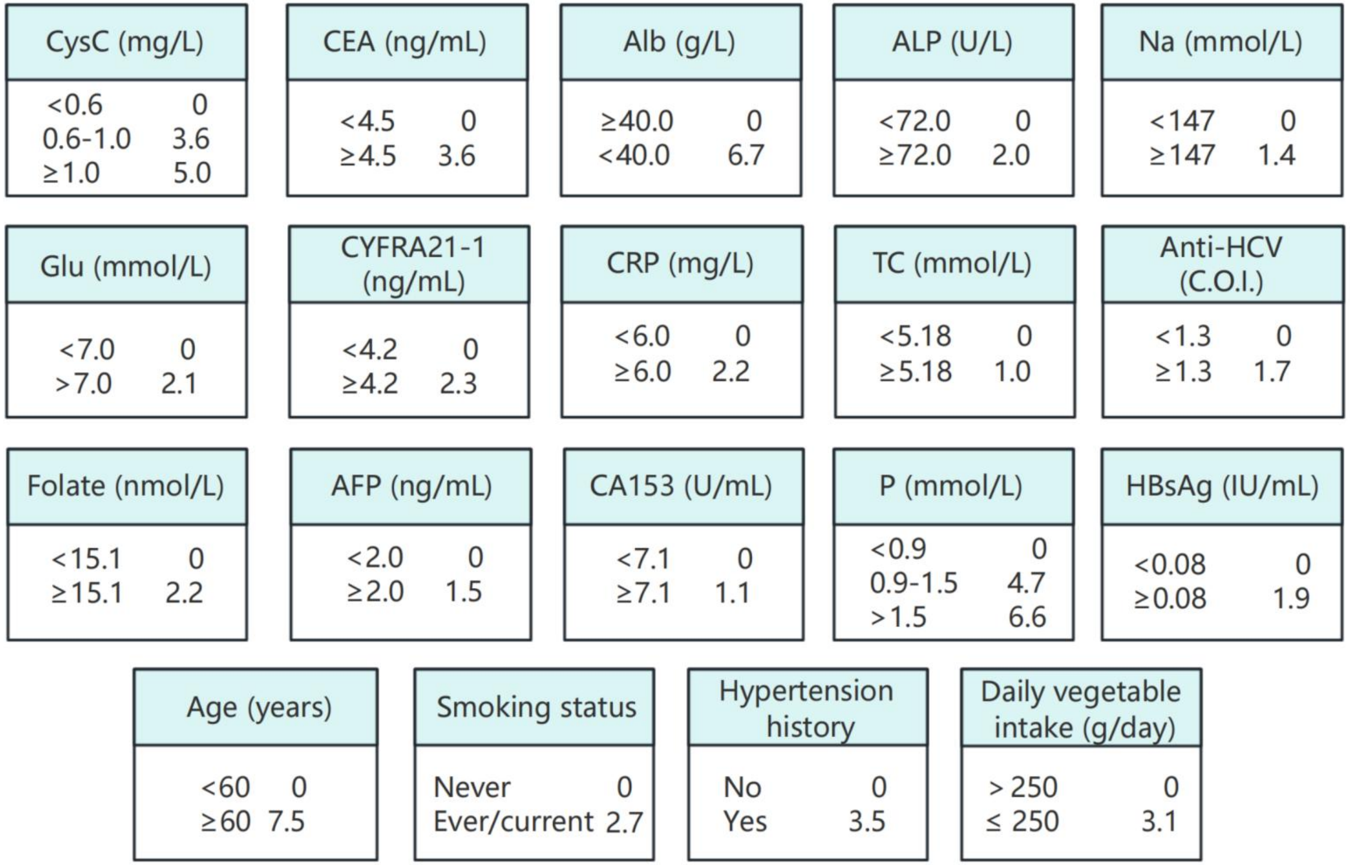

Legend: Points were derived from Cox regression coefficients and multiplied by 10 for display and interpretability. Biomarker abbreviations are defined in Table S1.

Figure S5: Risk stratification performance of the main score in the discovery cohort (Taizhou, China, 2011–2023)

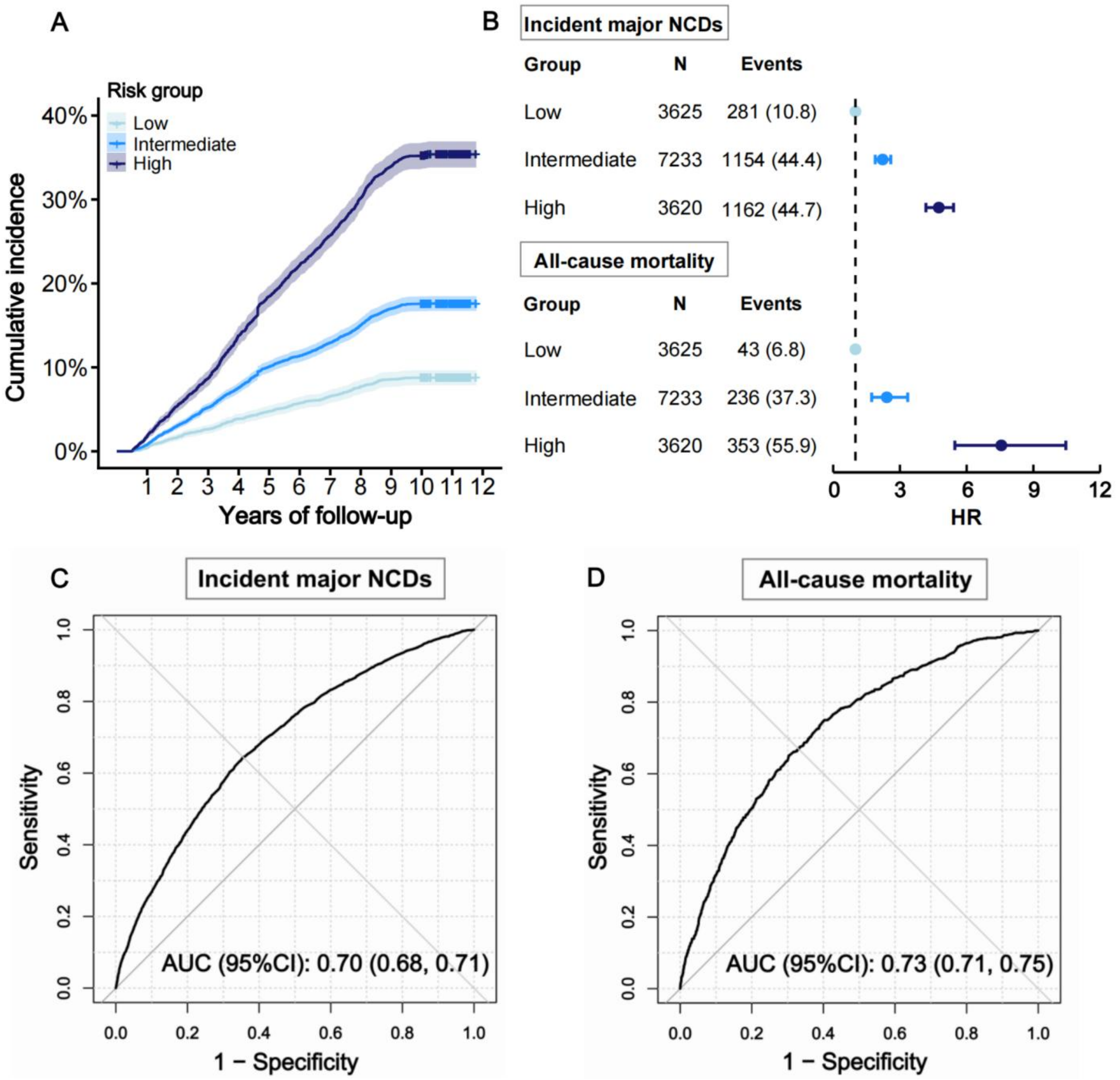

Legend: (A) Cumulative incidence of incident major NCDs by risk group. (B) HRs and 95% CIs for incident major NCDs and all-cause mortality by risk group. Percentages indicate the proportion of all events captured by each risk group. (C–D) Receiver operating characteristic curves for incident major NCDs and all-cause mortality.

Abbreviations: AUC, area under the receiver operating characteristic curve; CI, confidence interval; HR, hazard ratio; NCDs, non-communicable diseases.

Figure S6: Subgroup and sensitivity analyses for the main score (Taizhou, China, 2011–2023)

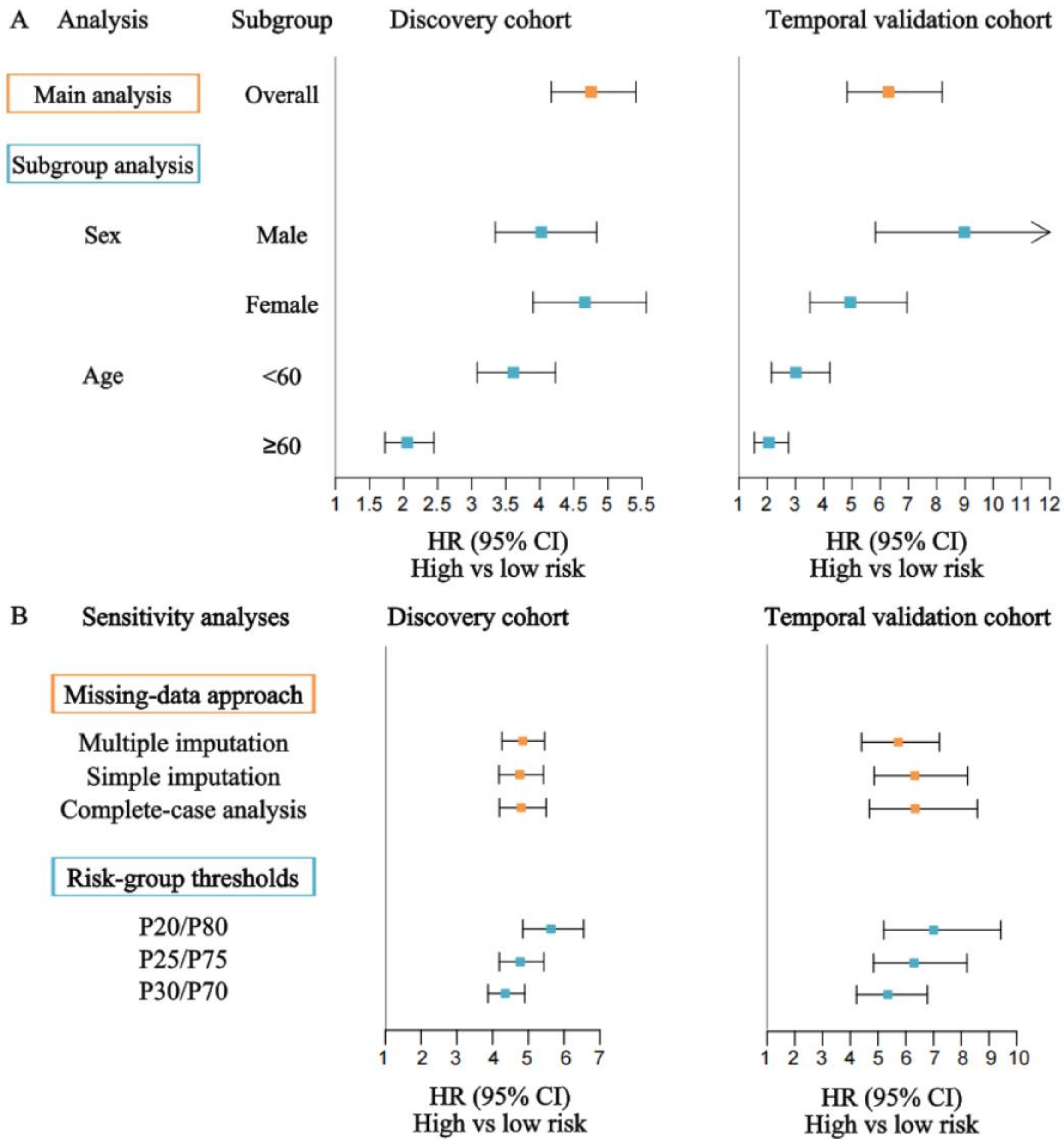

Legend: Hazard ratios and 95% confidence intervals were estimated for the high-risk group compared with the low-risk group. Subgroup analyses were conducted by sex and age. Sensitivity analyses compared alternative missing-data approaches and risk-group thresholds. Arrows indicate confidence intervals extending beyond the plotting range. P20/P80, P25/P75, and P30/P70 indicate percentile-based risk-group thresholds.

Table S5: Sensitivity analyses using alternative modeling strategies for the main score (Taizhou, China, 2011–2023)

Panel A. Alternative predictor-selection strategies

Model	Predictors, n	Discovery cohort			Temporal validation cohort		
		HR (95% CI)	AUC (95% CI)	high-risk events, n (%)	HR (95% CI)	AUC (95% CI)	high-risk events, n (%)
Main score	19	4.75 (4.17, 5.41)	0.70 (0.68, 0.71)	1162 (44.7)	6.29 (4.83, 8.19)	0.70 (0.68, 0.72)	391 (53.9)
Excluding-stepwise consensus	25	5.47 (4.77, 6.27)	0.70 (0.68, 0.71)	1177 (45.3)	6.06 (4.66, 7.88)	0.70 (0.68, 0.72)	383 (52.8)
Majority-vote	28	5.41 (4.72, 6.20)	0.70 (0.68, 0.71)	1179 (45.4)	6.26 (4.79, 8.17)	0.70 (0.68, 0.71)	383 (52.8)
Union model	35	5.38 (4.70, 6.16)	0.70 (0.68, 0.71)	1182 (45.5)	6.38 (4.88, 8.34)	0.69 (0.68, 0.71)	384 (52.9)

Panel B. Alternative biomarker processing

Biomarker form	Predictors, n	Discovery cohort			Temporal validation cohort		
		HR (95% CI)	AUC (95% CI)	high-risk events, n (%)	HR (95% CI)	AUC (95% CI)	high-risk events, n (%)
Categorical	19	4.75 (4.17, 5.41)	0.70 (0.68, 0.71)	1162 (44.7)	6.29 (4.83, 8.19)	0.70 (0.68, 0.72)	391 (53.9)
Continuous z-score	19	5.33 (4.66, 6.11)	0.70 (0.68, 0.71)	1179 (45.4)	6.49 (4.96, 8.48)	0.70 (0.68, 0.72)	390 (53.7)

Note: HRs compare the high-risk group with the low-risk group. High-risk events indicate the number and proportion of incident major NCD events occurring in the high-risk group. The excluding-stepwise consensus score included predictors selected by both LASSO and Boruta. The majority-vote score included predictors selected by at least two of LASSO, stepwise regression, and Boruta. The union score included predictors selected by any of the three methods. Categorical indicates cut-off-based point assignment. Continuous z-score retained the final predictors and recalculated the score using z-standardized circulating blood biomarkers. Abbreviations: NCDs, non-communicable diseases; HR, hazard ratio; CI, confidence interval; AUC, area under the receiver operating characteristic curve; LASSO, least absolute shrinkage and selection operator.

Table S6: Outcome and follow-up window sensitivity analyses (Taizhou, China, 2011–2023)

Panel A. Expanded outcome definitions and glucose-excluded score

Outcome definition	Score	Cohort	Events	HR (95% CI)	AUC (95% CI)	High-risk events, n (%)
Original major NCDs	Main score	Discovery	2,597	4.75 (4.17, 5.41)	0.70 (0.68, 0.71)	1162 (44.7)
		Temporal validation	726	6.29 (4.83, 8.19)	0.70 (0.68, 0.72)	391 (53.9)
Expanded major NCDs	Main score	Discovery	3,262	4.10 (3.67, 4.58)	0.69 (0.67, 0.70)	1393 (42.7)
		Temporal validation	1,343	6.07 (4.99, 7.38)	0.70 (0.68, 0.71)	682 (50.8)
	Score excluding glucose	Discovery	3,262	3.76 (3.37, 4.19)	0.67 (0.66, 0.68)	1369 (42.0)
		Temporal validation	1,343	4.22 (3.44, 5.18)	0.67 (0.66, 0.69)	748 (55.7)

Panel B. Fixed 3-year follow-up window for incident major non-communicable diseases

Cohort	Risk group	N	Events	3-year absolute risk, %	Event concentration, %	HR (95% CI)
Discovery	Low	3,625	84	2.32	11.3	1.00
	Intermediate	7,233	352	4.87	47.2	2.13 (1.68, 2.70)
	High	3,620	310	8.56	41.6	3.82 (3.00, 4.87)
Temporal validation	Low	6,257	63	1.01	8.9	1.00
	Intermediate	12,509	262	2.10	37.1	2.09 (1.59, 2.76)
	High	6,252	382	6.14	54.0	6.23 (4.77, 8.13)

Note: In Panel A, HRs compare the high-risk group with the low-risk group. In Panel B, HRs compare each risk group with the low-risk group. High-risk events indicate the number and proportion of events occurring in the high-risk group. The expanded major NCD outcome additionally included incident diabetes, breast cancer, and asthma. The glucose-excluded score was recalculated after removing the glucose component from the main score without repeating predictor selection. In the fixed 3-year analysis, follow-up was restricted to 3 years after baseline, and absolute risks were estimated at 3 years. Event concentration indicates the proportion of events occurring in each risk group. Abbreviations: NCDs, non-communicable diseases; HR, hazard ratio; CI, confidence interval; AUC, area under the receiver operating characteristic curve.

Table S7: Event composition of the composite outcome and performance of the main score across outcome definitions (Taizhou, China, 2011–2023)

Panel A. Distribution of first event types within the original composite outcome

Cohort	Original major NCD events	First cancer, n (%)	First CVD, n (%)	First CRD, n (%)	Cancer + CRD, n (%)
Discovery cohort	2,597	460 (17.7)	1,507 (58.0)	630 (24.3)	1,090 (42.0)
Temporal validation cohort	726	171 (23.6)	437 (60.2)	118 (16.3)	289 (39.8)

Panel B. Performance of the NIMO score across outcome definitions

Cohort	Outcome evaluated	Events	High-risk events, n (%)	High vs low HR (95% CI)	AUC (95% CI)
Discovery cohort	Original major NCDs	2,597	1,162 (44.7)	4.75 (4.17, 5.41)	0.70 (0.68, 0.71)
	Non-CVD	1,090	457 (41.9)	4.44 (3.62, 5.44)	0.66 (0.64, 0.67)
	Cancer	460	228 (49.6)	7.02 (4.98, 9.90)	0.71 (0.68, 0.72)
	CVD	1,507	705 (46.8)	4.98 (4.20, 5.90)	0.68 (0.67, 0.69)
	CRD	630	229 (36.3)	3.22 (2.50, 4.16)	0.62 (0.60, 0.64)
Temporal validation cohort	Original major NCDs	726	391 (53.9)	6.29 (4.83, 8.19)	0.70 (0.68, 0.72)
	Non-CVD	289	157 (54.3)	6.23 (4.11, 9.43)	0.70 (0.67, 0.73)
	Cancer	171	91 (53.2)	7.84 (4.29, 14.32)	0.71 (0.68, 0.75)
	CVD	437	234 (53.5)	6.33 (4.49, 8.92)	0.70 (0.67, 0.72)
	CRD	118	66 (55.9)	4.85 (2.72, 8.63)	0.68 (0.63, 0.73)

Note: Panel A shows the distribution of first event types within the original major NCD composite outcome. Panel B shows the performance of the NIMO score across alternative outcome definitions. The Non-CVD category was defined as first incident cancer or CRD. HRs compare the high-risk group with the low-risk group. High-risk events indicate the number and proportion of events occurring in the high-risk group. Abbreviations: NIMO, Major NCDs Identification Model; NCDs, non-communicable diseases; CVD, cardio-cerebrovascular diseases; CRD, chronic respiratory diseases; HR, hazard ratio; CI, confidence interval; AUC, area under the receiver operating characteristic curve.

Table S8: Internal robustness analyses of the main score (Taizhou, China, 2011–2023)

Panel A. Repeated 7:3 internal validation

Analysis	AUC	Intermediate vs low HR	High vs low HR	High-risk event concentration, %
Training subset, median (range)	0.70 (0.69–0.71)	2.24 (2.02–2.53)	5.07 (4.57–5.77)	45.5 (43.3–47.2)
Testing subset, median (range)	0.69 (0.68–0.72)	2.15 (1.64–2.57)	4.79 (3.73–6.05)	45.2 (39.6–48.8)

Panel B. Bootstrap variable-selection stability of the selected predictors

Predictor	LASSO (%)	Stepwise (%)	Boruta (%)	All-three consensus (%)
Age	100	100	100	100
Smoking	100	100	100	100
Hypertension	100	100	100	100
Daily vegetable intake	100	100	100	100
AFP	100	96	100	96
CEA	100	100	100	100
CA153	100	98	100	98
CYFRA21-1	100	84	100	84
CRP	100	86	100	86
HBsAg	100	98	100	98
Anti-HCV	98	80	100	80
Alb	100	98	100	98
ALP	100	100	100	100
CysC	100	100	100	100
Glu	100	92	100	92
TC	100	90	100	90
P	98	88	100	88
Na	100	82	100	82
Folate	100	96	100	96

Note: Panel A shows repeated 7:3 internal validation within the discovery cohort. In each of 50 repeats, the final predictors of the main score were retained, Cox coefficients were re-estimated in the 70% training subset, and model performance was evaluated in both the training subset and the 30% testing subset. Values in Panel A are presented as median (range). Panel B shows variable-selection stability based on repeating the full predictor-selection procedure in 50 bootstrap resamples of the discovery cohort. Percentages indicate selection frequencies. HRs compare the intermediate- or high-risk group with the low-risk group. High-risk event concentration indicates the proportion of incident major NCD events occurring in the high-risk group. Biomarker abbreviations are defined in Table S1. Abbreviations: NCDs, non-communicable diseases; AUC, area under the receiver operating characteristic curve; HR, hazard ratio; LASSO, least absolute shrinkage and selection operator.

Table S9: Predictor sources and selected predictors for the main, disease-specific, and information-source scores (Taizhou, China, 2011–2023)

Score	Predictor source	Selected predictors
Main score	Traditional + biomarkers	Age, smoking, hypertension, daily vegetable intake, AFP, CEA, CA153, CYFRA21-1, CRP, HBsAg, Anti-HCV, Alb, ALP, CysC, Glu, TC, P, Na, Folate
Cancer-specific score	Traditional + biomarkers	Age, sex, smoking, alcohol drinking, daily vegetable intake, AFP, CEA, CYFRA21-1, Alb, AST, HDL, TP, CysC, Na, P, CRP, HBsAg
CVD-specific score	Traditional + biomarkers	Age, smoking, hypertension, daily vegetable intake, CEA, CYFRA21-1, Ferritin, TC, CysC, CRP, Glu, Amy, Folate
CRD-specific score	Traditional + biomarkers	Age, daily vegetable intake, CEA, CA153, CYFRA21-1, ALP, AST, DBIL, TG, CysC, P, CRP, CO_2, Folate
Traditional-factor score	Traditional only	Age, sex, education, marital status, smoking, hypertension, daily vegetable intake
Blood-based score	Biomarkers + age/sex	Age, sex, AFP, CEA, CA153, CYFRA21-1, HBsAg, Anti-HCV, Alb, ALP, CysC, Glu, TC, P, Na, Folate, GGT, CRP

Note: The main score refers to the NIMO score. Traditional factors were questionnaire- and physical examination-derived variables; biomarkers were circulating blood biomarkers. Biomarker abbreviations are defined in Table S1. Abbreviations: NIMO, Major NCDs Identification Model; CVD, cardio-cerebrovascular diseases; CRD, chronic respiratory diseases.

Table S10: Risk stratification performance of the main, traditional-factor, and blood-based scores (Taizhou, China, 2011–2023)

Score	Risk group	Discovery cohort				Temporal validation cohort			
		N	Events, n (%)	HR (95% CI)	P for trend	N	Events, n (%)	HR (95% CI)	P for trend
Incident major NCDs									
Main score	Low	3,625	281 (7.8%)	1.00	**<0.001**	6,257	64 (1.0%)	1.00	**<0.001**
	Intermediate	7,233	1,154 (16.0%)	2.16 (1.89, 2.46)		12,509	271 (2.2%)	2.13 (1.62, 2.80)	
	High	3,620	1,162 (32.1%)	4.75 (4.17, 5.41)		6,252	391 (6.3%)	6.29 (4.83, 8.19)	
Traditional-factor score	Low	3,951	434 (11.0%)	1.00	**<0.001**	6,255	105 (1.7%)	1.00	**<0.001**
	Intermediate	7,174	1,085 (15.1%)	1.41 (1.26, 1.58)		12,509	242 (1.9%)	1.16 (0.92, 1.46)	
	High	3,353	1,078 (32.2%)	3.30 (2.95, 3.69)		6,254	379 (6.1%)	3.71 (2.99, 4.61)	
Blood-based score	Low	3,639	282 (7.7%)	1.00	**<0.001**	6,977	87 (1.2%)	1.00	**<0.001**
	Intermediate	7,220	1,153 (16.0%)	2.16 (1.89, 2.46)		11,786	259 (2.2%)	1.77 (1.39, 2.26)	
	High	3,619	1,162 (32.1%)	4.74 (4.16, 5.40)		6,255	380 (6.1%)	5.00 (3.96, 6.31)	
All-cause mortality									
Main score	Low	3,625	43 (1.2%)	1.00	**<0.001**	6,257	10 (0.2%)	1.00	**<0.001**
	Intermediate	7,233	236 (3.3%)	2.41 (1.73, 3.35)		12,509	76 (0.6%)	3.48 (1.80, 6.73)	
	High	3,620	353 (9.8%)	7.56 (5.47, 10.46)		6,252	127 (2.0%)	12.74 (6.68, 24.30)	
Traditional-factor score	Low	3,951	86 (2.2%)	1.00	**<0.001**	6,255	19 (0.3%)	1.00	**<0.001**
	Intermediate	7,174	211 (2.9%)	1.29 (1.01, 1.66)		12,509	73 (0.6%)	3.02 (1.80, 5.06)	
	High	3,353	335 (10.0%)	4.52 (3.56, 5.73)		6,254	121 (1.9%)	9.20 (5.62, 15.06)	
Blood-based score	Low	3,639	47 (1.3%)	1.00	**<0.001**	6,977	16 (0.2%)	1.00	**<0.001**
	Intermediate	7,220	224 (3.1%)	2.05 (1.48, 2.84)		11,786	60 (0.5%)	1.92 (1.09, 3.37)	
	High	3,619	361 (10.0%)	7.02 (5.11, 9.65)		6,255	137 (2.2%)	8.89 (5.21, 15.20)	

Note: Percentages in parentheses indicate the event proportion within each risk group. HRs and 95% CIs were estimated using Cox proportional hazards regression models, with the low-risk group as the reference. P for trend indicates the trend test across low-, intermediate-, and high-risk groups. Abbreviations: HR, hazard ratio; CI, confidence interval; NCDs, non-communicable diseases.

Figure S7: Discrimination of the main, traditional-factor, and blood-based scores (Taizhou, China, 2011–2023)

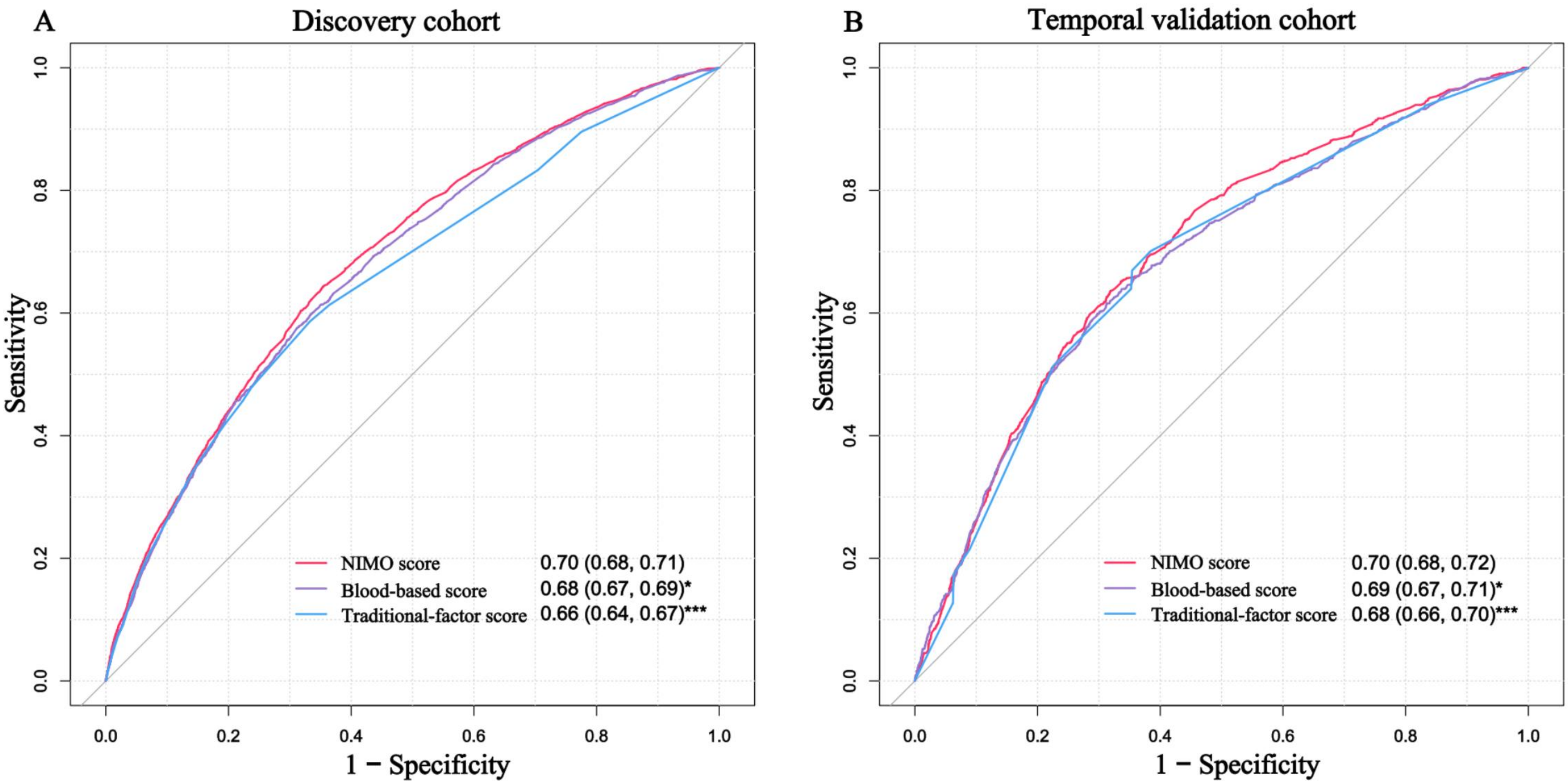

Legend: Receiver operating characteristic curves compare the NIMO score with blood-based and traditional-factor scores for incident major NCDs. Values indicate AUCs with 95% CIs. Asterisks indicate DeLong test results compared with the NIMO score: *P < 0.05; ***P < 0.001. Abbreviations: NIMO, Major NCDs Identification Model; NCDs, non-communicable diseases; AUC, area under the receiver operating characteristic curve; CI, confidence interval.

Supplementary References

S1. WHO CVD Risk Chart Working Group. World Health Organization cardiovascular disease risk charts: revised models to estimate risk in 21 global regions. Lancet Glob Health (2019) 7:e1332–45. doi: 10.1016/S2214-109X(19)30318-3

S2. Han S, Mo G, Gao T, Sun Q, Liu H, Zhang M. Age, sex, residence, and region-specific differences in prevalence and patterns of multimorbidity among older Chinese: evidence from Chinese Longitudinal Healthy Longevity Survey. BMC Public Health (2022) 22:1116. doi: 10.1186/s12889-022-13506-0

S3. He H, Pan Z, Wu J, Hu C, Bai L, Lyu J. Health effects of tobacco at the global, regional, and national levels: results from the 2019 Global Burden of Disease Study. Nicotine Tob Res (2022) 24:864–70. doi: 10.1093/ntr/ntab265

S4. Unger T, Borghi C, Charchar F, Khan NA, Poulter NR, Prabhakaran D, et al. 2020 International Society of Hypertension global hypertension practice guidelines. Hypertension (2020) 75:1334–57. doi: 10.1161/HYPERTENSIONAHA.120.15026

S5. Sionakidis A, McCallum L, Padmanabhan S. Unravelling the tangled web of hypertension and cancer. Clin Sci (Lond) (2021) 135:1609–25. doi: 10.1042/CS20200307

S6. Aune D, Giovannucci E, Boffetta P, Fadnes LT, Keum N, Norat T, et al. Fruit and vegetable intake and the risk of cardiovascular disease, total cancer and all-cause mortality: a systematic review and dose-response meta-analysis of prospective studies. Int J Epidemiol (2017) 46:1029–56. doi: 10.1093/ije/dyw319

S7. Bracun V, Suthahar N, Shi C, de Wit S, Meijers WC, Klip IT, et al. Established tumour biomarkers predict cardiovascular events and mortality in the general population. Front Cardiovasc Med (2021) 8:753885. doi: 10.3389/fcvm.2021.753885

S8. Hanif H, Ali MJ, Susheela AT, Khan IW, Luna-Cuadros MA, Khan MM, et al. Update on the applications and limitations of alpha-fetoprotein for hepatocellular carcinoma. World J Gastroenterol (2022) 28:216–29. doi: 10.3748/wjg.v28.i2.216

S9. Sölétormos G, Nielsen D, Schiøler V, Mouridsen H, Dombernowsky P. Monitoring different stages of breast cancer using tumour markers CA 15-3, CEA and TPA. Eur J Cancer (2004) 40:481–6. doi: 10.1016/j.ejca.2003.10.015

S10. Fahim A, Crooks MG, Wilmot R, Campbell AP, Morice AH, Hart SP. Serum carcinoembryonic antigen correlates with severity of idiopathic pulmonary fibrosis. Respirology (2012) 17:1247–52. doi: 10.1111/j.1440-1843.2012.02231.x

S11. Shimada H, Noie T, Ohashi M, Oba K, Takahashi Y. Clinical significance of serum tumor markers for gastric cancer: a systematic review of literature by the Task Force of the Japanese Gastric Cancer Association. Gastric Cancer (2014) 17:26–33. doi: 10.1007/s10120-013-0259-5

S12. Ajona D, Remirez A, Sainz C, Bertolo C, Gonzalez A, Varo N, et al. A model based on the quantification of complement C4c, CYFRA 21-1 and CRP exhibits high specificity for the early diagnosis of lung cancer. Transl Res (2021) 233:77–91. doi: 10.1016/j.trsl.2021.02.009

S13. Heo J, Moon DH, Hong Y, Bak SH, Kim J, Park JH, et al. Word embedding reveals Cyfra 21-1 as a biomarker for chronic obstructive pulmonary disease. J Korean Med Sci (2021) 36:e224. doi: 10.3346/jkms.2021.36.e224

S14. Hao B, Liu Y, Wang B, Wu H, Chen Y, Zhang L. Hepatitis B surface antigen: carcinogenesis mechanisms and clinical implications in hepatocellular carcinoma. Exp Hematol Oncol (2025) 14:44. doi: 10.1186/s40164-025-00642-7

S15. de Martel C, Georges D, Bray F, Ferlay J, Clifford GM. Global burden of cancer attributable to infections in 2018: a worldwide incidence analysis. Lancet Glob Health (2020) 8:e180–90. doi: 10.1016/S2214-109X(19)30488-7

S16. Lee KK, Stelzle D, Bing R, Anwar M, Strachan F, Bashir S, et al. Global burden of atherosclerotic cardiovascular disease in people with hepatitis C virus infection: a systematic review, meta-analysis, and modelling study. Lancet Gastroenterol Hepatol (2019) 4:794–804. doi: 10.1016/S2468-1253(19)30227-4

S17. Emerging Risk Factors Collaboration, Kaptoge S, Di Angelantonio E, Lowe G, Pepys MB, Thompson SG, et al. C-reactive protein concentration and risk of coronary heart disease, stroke, and mortality: an individual participant meta-analysis. Lancet (2010) 375:132–40. doi: 10.1016/S0140-6736(09)61717-7

S18. Zhu M, Ma Z, Zhang X, Hang D, Yin R, Feng J, et al. C-reactive protein and cancer risk: a pan-cancer study of prospective cohort and Mendelian randomization analysis. BMC Med (2022) 20:301. doi: 10.1186/s12916-022-02506-x

S19. Kühn T, Sookthai D, Graf ME, Schübel R, Freisling H, Johnson T, et al. Albumin, bilirubin, uric acid and cancer risk: results from a prospective population-based study. Br J Cancer (2017) 117:1572–9. doi: 10.1038/bjc.2017.313

S20. Lan CC, Su WL, Yang MC, Chen SY, Wu YK. Predictive role of neutrophil-percentage-to-albumin, neutrophil-to-lymphocyte and eosinophil-to-lymphocyte ratios for mortality in patients with COPD: evidence from NHANES 2011–2018. Respirology (2023) 28:1136–46. doi: 10.1111/resp.14589

S21. Wannamethee SG, Sattar N, Papcosta O, Lennon L, Whincup PH. Alkaline phosphatase, serum phosphate, and incident cardiovascular disease and total mortality in older men. Arterioscler Thromb Vasc Biol (2013) 33:1070–6. doi: 10.1161/ATVBAHA.112.300826

S22. Wu Q, Wang Y, Wei X, Xia J, Zhou K, Wang Y, et al. Association between alkaline phosphatase and cancer in American adults: 2003–2016 NHANES. BMC Cancer (2025) 25:1157. doi: 10.1186/s12885-025-14533-x

S23. Mo D, Zhang P, Zhang M, Dai H, Guan J. Cholesterol, high-density lipoprotein, and glucose index versus triglyceride-glucose index in predicting cardiovascular disease risk: a cohort study. Cardiovasc Diabetol (2025) 24:116. doi: 10.1186/s12933-025-02675-y

S24. Wang T, Zhu Y, Liu X, Zhang Y, Zhang Z, Wu J, et al. Cystatin C and sarcopenia index are associated with cardiovascular and all-cause death among adults in the United States. BMC Public Health (2024) 24:1972. doi: 10.1186/s12889-024-19137-x

S25. Chai L, Feng W, Zhai C, Shi W, Wang J, Yan X, et al. The association between cystatin C and COPD: a meta-analysis and systematic review. BMC Pulm Med (2020) 20:182. doi: 10.1186/s12890-020-01208-5

S26. Emerging Risk Factors Collaboration, Sarwar N, Gao P, Seshasai SR, Gobin R, Kaptoge S, et al. Diabetes mellitus, fasting blood glucose concentration, and risk of vascular disease: a collaborative meta-analysis of 102 prospective studies. Lancet (2010) 375:2215–22. doi: 10.1016/S0140-6736(10)60484-9

S27. Wang M, Liu J, Yan L, Wang J, Jin Y, Zheng ZJ. Burden of liver cancer attributable to high fasting plasma glucose: a global analysis based on the Global

Burden of Disease Study 2019. J Nutr Health Aging (2024) 28:100261. doi: 10.1016/j.jnha.2024.100261

S28. Takata Y, Shrubsole MJ, Li H, Cai Q, Gao J, Wagner C, et al. Plasma folate concentrations and colorectal cancer risk: a case-control study nested within the Shanghai Men's Health Study. Int J Cancer (2014) 135:2191–8. doi: 10.1002/ijc.28871

S29. Zhao Q, Lv X, Liu Q, Hu Z, Zhan Y. Association between serum folate concentrations and all-cause mortality in U.S. adults: a cohort study based on National Health and Nutrition Examination Survey III. Front Nutr (2024) 11:1408023. doi: 10.3389/fnut.2024.1408023

S30. Dmitrieva NI, Gagarin A, Liu D, Wu CO, Boehm M. Middle-age high normal serum sodium as a risk factor for accelerated biological aging, chronic diseases, and premature mortality. EBioMedicine (2023) 87:104404. doi: 10.1016/j.ebiom.2022.104404

S31. Torrijo-Belanche C, Moreno-Franco B, Muñoz-Cabrejas A, Calvo-Galiano N, Casasnovas JA, Sayón-Orea C, et al. High serum phosphate is associated with cardiovascular mortality and subclinical coronary atherosclerosis: systematic review and meta-analysis. Nutrients (2024) 16:1599. doi: 10.3390/nu16111599

S32. Campos-Obando N, Lahousse L, Brusselle G, Stricker BH, Hofman A, Franco OH, et al. Serum phosphate levels are related to all-cause, cardiovascular and COPD mortality in men. Eur J Epidemiol (2018) 33:859–71. doi: 10.1007/s10654-018-0407-7
